# Comparison of Corneal Parameters of Children with Diabetes Mellitus and Healthy Children

**DOI:** 10.1155/2019/2037072

**Published:** 2019-11-04

**Authors:** Shanshan Wang, Yan Jia, Tao Li, Anken Wang, Lu Gao, Chenhao Yang, Haidong Zou

**Affiliations:** ^1^Department of Ophthalmology, Shanghai General Hospital (Shanghai First People's Hospital), Shanghai Jiao Tong University School of Medicine, Shanghai, China; ^2^Shanghai Key Laboratory of Fundus Diseases, Shanghai, China; ^3^Shanghai Engineering Center for Visual Science and Photomedicine, Shanghai, China; ^4^Shanghai Eye Diseases Prevention & Treatment Center, Shanghai Eye Hospital, Shanghai, China; ^5^Department of Ophthalmology, Children's Hospital of Fudan University, Shanghai, China

## Abstract

**Purpose:**

To compare differences in central corneal thickness (CCT), corneal curvature, and other corneal measurements of children with diabetes mellitus (DM) and healthy children, and to investigate related factors.

**Methods:**

This was a case-control study. From January to February 2018, 50 children with diabetes mellitus were selected as a case group, and 46 healthy children and adolescents without diabetes mellitus were selected as a control group. Corneal topography and CCT were analyzed using a corneal topography measuring apparatus and biometrics (IOL Master). In the diabetic group, we analyzed whether age, course of disease, sex, glycosylated hemoglobin, triglyceride level, total cholesterol, body mass index (BMI), parental BMI, birth history, feeding history, pregnancy, or puerperal history were related to corneal morphology.

**Results:**

There was a significant difference in CCT between groups, but no significant differences were found in corneal diameter, corneal curvature R1 or R2, or corneal topography. Central corneal thickness was not correlated with other clinical factors in the diabetes group.

**Conclusion:**

Early screening and close follow-up of keratopathy in children with diabetes are imperative.

## 1. Introduction

The global prevalence of diabetes is increasing rapidly. According to the 8th edition of the Global Diabetes Map released by the International Diabetes Federation, about 425 million adults worldwide suffered from diabetes in 2017. This number is expected to reach 629 million by 2045 [1]. The age of onset of diabetes is also becoming increasingly lower. The incidence of diabetes in adolescents and children is increasing globally [2]. In 2017, the number of children and adolescents (<20 years old) with type 1 diabetes reached 1,106,500. Among them, approximately 47,000 were Chinese patients, with China ranking the 4^th^ in the world for childhood diabetes [3]. Epidemiological studies conducted in China showed that, in 2010, the number of people with type 1, type 2, and other types of diabetes per 100,000 children and adolescents was 96.8, 8.0, and 3.3, respectively. This total, of about 286,000, demonstrates a significant upward trend in the last 15 years [4].

Diabetes and its associated chronic complications are a heavy burden to patients, families, and society [3]. Diabetes can cause ocular surface diseases (including dry eye and corneal disease), retinopathy, glaucoma, cataracts, refractive abnormalities, and other complications which can lead to significant visual loss or even blindness [4]. At present, there are many studies on corneal morphology and related factors in adults with diabetes mellitus patients [5–9] but few on corneal morphology in children with diabetes mellitus. With the increasing number of children and adolescents with diabetes mellitus, the results of corneal change, and risk factors in this population are of social significance and becoming more and more valuable. This paper describes a case-control study conducted in Shanghai, China, with the aim of providing reference data for the prevention and treatment of keratopathy in children and adolescents with diabetes mellitus.

## 2. Materials and Methods

This was a hospital-based case-control study (clinicaltrials.gov identifier: NCT03587948). It is a part of Shanghai Children and Adolescent Diabetes Eye study (SCADE) [10]. From January 2018 to February 2018, children who were diagnosed with diabetes at Children's Hospital of Fudan University in Shanghai were recruited. We also recruited age- and gender-matched controls without diabetes from the same hospital. The inclusion criteria were as follows: (1) provision of a written informed consent by a guardian, (2) age over 5 years and less than 18 years, and (3) diagnosis of type 1 or type 2 diabetes based on the WHO diagnostic criteria for diabetes. The exclusion criteria were eye diseases or other systemic diseases. These included (1) eyelid diseases—eyelid entropion, eyelid ectropion, eyelid, ptosis, and palpebral dyskinesia; (2) conjunctival diseases—pterygium and conjunctivitis; (3) history of chemical eye injuries; (4) history of eye surgeries within 6 months or history of retinal laser photocoagulation; (5) systemic diseases such as Sjogren syndrome, juvenile rheumatoid arthritis, Grave's disease, or juvenile-onset systemic lupus erythematosus.

Participation in the study was voluntary for both participants and their guardians, and all signed informed consent after the purpose and process of the study was explained in detail.

The research team comprised 3 ophthalmologists at the Shanghai General Hospital and Children's Hospital of Fudan University in Shanghai, 3 optometrists, and 15 auxiliary staff. The ophthalmologist with experience in the diagnosis of ocular diseases and knowledge of their epidemiology was the project director.

The examination was conducted in the ophthalmology clinic at Children's Hospital of Fudan University in Shanghai. We followed the methods of Li et al. [11]. First, baseline characteristics and subjective symptoms were surveyed using a questionnaire. The basic information collected included age, sex, history of diabetes, medication history, family medical history, parental history and refractive power (measured in diopters), birth and feeding history, current height and weight of children and their parents, mother's weight before pregnancy, mother's maximum weight during pregnancy, and mother's postpartum weight (weight of the mother within one month after child birth). The questionnaires were completed by the subjects and their guardians.

Next, the eye examination was performed. The examination comprised several tests. First, an ophthalmic slit lamp biomicroscopy (SL130, Zeiss, Germany) examination of the eyelids, conjunctiva, cornea, anterior chamber, iris, pupil, and lens was performed. Second, a corneal topographic examination (ATLAS, ZEISS, Germany) was performed. In this examination, the corneal morphology was analyzed digitally by a computer image processing system, and the corneal shape and regularity were analyzed as a whole. Third, biometry (IOL Master 700, Zeiss, Germany) was used to measure the axial length, lens thickness, pupil diameter, anterior chamber depth, and central corneal thickness (CCT). Fourth, a noncontact tonometer was used to measure the level of intraocular pressure (NT-510, Nidek, Japan).

Finally, within 6 months, the results of routine bloodwork were obtained at Children's Hospital of Fudan University in Shanghai.

Diabetes diagnoses (both type 1 and type 2) were based on the WHO diagnostic criteria [12], which are as follows: (1) typical symptoms (polydipsia, polyuria, and unexplained weight loss), fasting blood glucose > 7 mmol/l, or postprandial blood glucose > 11.1 mmol/l. (2) If no typical symptoms are present but fasting blood glucose > 7 mmol/l, or postprandial blood glucose > 11.1 mmol/l, the test is performed twice and those who still reach the above values are diagnosed with diabetes mellitus. (3) No typical symptoms with fasting blood glucose > 7 mmol/l, or postprandial glucose > 11.1 mmol/l, and glucose tolerance test 2-hour blood glucose > 11.1 mmol/l. These criteria are applied for both type 1 and type 2 diabetes.

On the corneal topographic map, we obtained five measures. (1) Steep K (D): the meridian direction and value of the maximum refractive power. Clinically, this represents the maximum curvature of the simulated cornea. (2) Flat K (D): the direction and value of the meridian line at an angle of 90 degrees with Steep K. Clinically, this represents the simulated curvature perpendicular to the maximum curvature of the cornea. (3) Astigmatism (D): an indication of corneal astigmatism. (4) Eccentricity (D): an indication of the change of refraction from the central cornea to the peripheral cornea. In clinical practice, the greater the difference between the central and peripheral corneal curvatures, the greater the corneal eccentricity (*E*-value). (5) *Q*: the aspheric coefficient. This represents the nonspherical nature and shape of the cornea along the meridian interface. Clinically, it signifies the degree of corneal curvature. *Q* = 0 represents a perfectly spherical surface. When *Q* > 0, it represents a flat center and a steep edge.

This survey was conducted by an investigation team that was trained by the project leader prior to the formal investigation. After the training, pretesting was performed in the investigation hospital, consistency was evaluated, and the measurement tools were tested. The project leader reviewed all the survey data after each investigation.

This study conformed to the ethical principles of the Helsinki declaration, and all participants provided written informed consent. The study was approved by the ethics committee of both Shanghai General Hospital (Approval No. 2016KY005), and Children's Hospital of Fudan University in Shanghai (Approval No. 01 (2018)).

Due to the high correlation between the two eyes of normal individuals, all ocular results were analyzed with right eye data.

In this study, BMI was calculated as body weight (kg) divided by height^2^ (m^2^). SPSS version 24 was used in statistical analysis. Measurement data are expressed as averages ± standard deviation, and categorical data are expressed as frequencies and percentages.

The Kolmogorov–Smirnov test was used to determine whether the measurement data conformed to the normal distribution. A chi-squared test or Student's *t* test was used if the distribution was normal; otherwise, a Mann-Whitney nonparametric test was used.

First, an independent samples *t*-test or Mann–Whitney nonparametric test was used to find significant differences in corneal topographic maps and corneal thickness values between the diabetic and the control group. Subsequently, Pearson correlation analysis was used to find clinical factors which were significantly correlated. A *P* value <0.05 was considered statistically significant.

## 3. Results

### 3.1. Demographic Comparisons

A total of 50 children with diabetes mellitus were enrolled in this study, including 48 patients with type 1 diabetes mellitus (96%) and 2 patients with type 2 diabetes mellitus (4%). Another 46 healthy subjects participated in the study as a control group. There were no significant differences in gender, age, birth weight, birth length, or BMI between groups (*P* > 0.05) (Table 1).

### 3.2. Comparison of Corneal Morphological Parameters

The differences in CCT, corneal diameter, corneal curvature R1 and R2, and corneal topography between groups were analyzed. There was a significant difference in CCT between groups (*P* < 0.05) (Table 2).

### 3.3. Correlation between CCT Value and Other Clinical Factors in Diabetes Group

Further analysis of the correlation between individual factor data and CCT value in the diabetes group showed no significant correlation between CCT value and individual factors (*P* > 0.05) (Table 3).

## 4. Discussion

This is the first study in China to examine corneal morphological changes in children with diabetes. There are few reports on corneal morphologic changes in children with diabetes. According to the results of a PUBMED search, there are currently 4 foreign studies [13–16] exploring the changes of corneal morphology and its risk factors in children and adolescents with diabetes mellitus. Among the 4 studies, the CCT value of the diabetes mellitus group was higher than that of the control group. In this study, we found that the CCT value of the diabetic group was significantly lower than that of the normal group (*P* < 0.05). In addition, we found no significant differences in R1, R2, or corneal diameter between the two groups. We believe that differences in race, age, duration of diabetes, endothelial cell density (ECD), and blood glucose control among children with diabetes mellitus may be the main reason for the differences in CCT results between studies in China and abroad.

A change in corneal thickness depends primarily on the degree of matrix dehydration [17], which relies on the maintenance of the corneal epithelial fluid barrier [18] and the dynamic balance of water and sodium diffusion between corneal endothelial cells and aqueous humor [19]. Diabetes mellitus can lead to corneal epithelial defect, reduced corneal endothelial cell reserve, morphological abnormalities, chronic corneal hypoxia, and chronic minimal injury [20]. Corneal epithelial cells are prone to hypoxia in the long-term high glucose environment, causing changes in the release of nucleotide and sputum signals [21]. Studies by Zagon et al. [22] have shown that the delay in epithelial re-formation depends on the course of diabetes and the involvement of endogenous opioid receptors, and opioid antagonists can promote the healing process of wounds. In addition, by using noncontact corneal endoscopic microscopy, researchers found that compared with normal human cornea, the density of corneal endothelial cells of patients with diabetes decreased and the percentage of normal hexagonal cells decreased [23]. Persistent hyperglycemia can stimulate the production of a large number of advanced glycation end products, activate and induce a series of inflammatory reactions, and generate a large number of reactive oxygen species causing further damage to corneal cells and resulting in apoptosis [24]. This may be the pathological mechanism behind thinner CCT in children with diabetes in this study.

Compared with foreign studies, we have collected a greater number of bodily factors, such as hematological examination results, parental status, family history, pregnancy and birth history, feeding history, and other factors related to the occurrence and development of children. However, we did not find factors related to CCT value. Unlike the current study, previous studies of adult diabetic patients showed that CCT values were correlated with duration of the disease [8] which is mainly because the longer the duration of diabetes, the greater the destruction of the corneal morphology. It is possible that we did not find this result due to the fact that the subjects in this study were children under 15 years of age and the duration of diabetes was relatively short. Further studies are still needed to explore the related factors of corneal morphologic changes in children with diabetes.

The limitations of this study are as follows: (1) The study was conducted at a single center with a small sample size. The subjects of this study are students who are busy during the semester, so we were only able to use winter vacation in 2018 to carry out the study. The participants signed informed consent before inclusion. 2. This study is a cross-sectional study. At present, we are preparing for a multicenter clinical study and follow-up observation.

In conclusion, this study found that CCT in children with diabetes mellitus was significantly lower than in healthy children and adolescents. Because children and adolescents lack the ability to accurately describe the subjective symptoms of the eye and cannot cooperate well during clinical examination, the incidence of keratopathy is easily overlooked. The long onset time has a significant negative effect on the ocular surface structure and eye health. Therefore, it is suggested that more attention should be paid to the corneal lesions of children with diabetes mellitus. Early screening and close follow-up of corneal diseases should be done and, once identified, treated in a timely manner.

## Figures and Tables

**Table 1:** A comparison of 50 children with diabetes mellitus and 46 normal controls enrolled in this study.

	Case group (*n* = 50)	Control group (*n* = 48)	Statistics	*P*
Age (years)	10.04 ± 2.70	9.43 ± 1.73	^§^ *t* = 1.645	0.103
Female	27	24	^‡^ *X* ^2^ = 1.93	0.165
Birth weight (kg)	3.37 ± 0.52	3.31 ± 0.46	*t* = 0.634	0.527
Body length at birth (cm)	50.70 ± 2.41	50.77 ± 2.87	*t* = −0.127	0.899
BMI (kg/m^2^)^†^	17.68 ± 3.95	17.10 ± 2.96	*t* = 0.808	0.421
Father's BMI (kg/m^2^)	24.73 ± 3.22	24.79 ± 3.33	*t* = −0.094	0.926
Mother's BMI (kg/m^2^)	21.87 ± 2.33	21.69 ± 2.36	*t* = 0.373	0.710
Prepregnancy BMI (kg/m^2^)	20.24 ± 1.85	20.22 ± 2.39	*t* = 0.043	0.966
Mother's maximum BMI^*∗∗*^ (kg/m^2^) during pregnancy	25.87 ± 2.96	25.32 ± 2.36	*t* = 0.982	0.329
Mother's BMI (kg/m^2^) within 1 month of delivery	22.60 ± 2.73	22.26 ± 2.35	*t* = 0.624	0.534
Gestational age (weeks)	38.24 ± 2.30	38.11 ± 1.39	*t* = 0.347	0.729

^†^BMI = body mass index = body weight (kg)/height^2^ (m^2^). ^‡^*X*^2^ = chi-squared test. ^§^*t* = independent samples *t* test.

**Table 2:** A comparison of corneal parameters between 50 children with diabetes mellitus and 46 normal controls enrolled in this study.

Corneal parameters	Case group (*n* = 50)	Control group (*n* = 48)	Statistics	*P*
Steep corneal curvature (D)	43.27 ± 1.37	43.21 ± 1.66	*t* ^†^ = 0.193	0.848
Flat corneal curvature (D)	42.16 ± 1.31	42.06 ± 1.57	*t* = 0.358	0.721
Astigmatism (D)	1.11 ± 0.53	1.15 ± 0.52	*t* = −0.397	0.692
Eccentricity (D)	0.62 ± 0.09	0.63 ± 0.10	*t* = −0.615	0.540
Corneal aspherical coefficient	−0.39 ± 0.11	−0.41 ± 0.12	*Z* ^‡^ = −0.446	0.656
Central corneal thickness (*μ*m)	562.27 ± 28.48	573.40 ± 32.94	*t* = −2.405	0.018
Corneal diameter (mm)	12.09 ± 0.41	11.66 ± 1.92	*Z* = −1.077	0.281
Corneal curvature R1 (D)	42.09 ± 1.80	42.04 ± 1.52	*t* = 0.131	0.896
Corneal curvature R2 (D)	43.12 ± 1.86	43.32 ± 1.68	*t* = −0.499	0.619
Intraocular pressure (mmHg)	18.21 ± 3.53	18.42 ± 2.61	*Z* = −0.402	0.688
Pupil diameter (mm)	5.46 ± 2.60	6.48 ± 2.34	*Z* = −1.941	0.052

^†^ *t* = Student's *t* test. ^‡^*Z* = Mann–Whitney nonparametric test.

**Table 3:** Correlation between CCT and individual factors in 50 children with diabetes mellitus enrolled in this study.

	Case group	Statistics	*P*
Duration of diabetes (years)	3.72 ± 0.27	*t* ^†^ = −0.048	0.742
Age (years)	10.04 ± 2.70	*t* = −0.209	0.146
Blood glycated hemoglobin (%)	7.58 ± 2.24	*t* = −0.077	0.600
Creatinine (mmol/l)	39.50 ± 8.89	*t* = −0.231	0.123
Blood triglyceride (mmol/l)	0.99 ± 1.05	*t* = 0.277	0.069
Total blood cholesterol (mmol/l)	4.60 ± 1.07	*t* = −0.025	0.874
Birth weight (kg)	3.37 ± 0.52	*t* = −0.048	0.742
Body length at birth (cm)	50.70 ± 2.41	*t* = 0.160	0.154
BMI^‡^ (kg/m^2^)	17.68 ± 3.95	*t* = −0.148	0.136
Prepregnancy BMI (kg/m^2^)	20.24 ± 1.85	*t* = −0.187	0.060
Mother's maximum BMI (kg/m^2^) during pregnancy	25.87 ± 2.96	*t* = −0.070	0.479
Mother's BMI (kg/m^2^) within 1 month of delivery	22.60 ± 2.73	*t* = −0.169	0.089
Gestational age (week)	38.24 ± 2.30	*t* = −0.001	0.993
Father's BMI (kg/m^2^)	24.73 ± 3.22	*t* = 0.062	0.529
Mother's BMI (kg/m^2^)	21.87 ± 2.33	*t* = −0.134	0.178
NCT^§^ (mmHg)	18.21 ± 3.53	*t* = 0.150	0.149
Corneal diameter (mm)	12.08 ± 0.41	*t* = −0.093	0.527
Pupil diameter (mm)	5.46 ± 2.60	*t* = 0.271	0.199

^†^ *t* = Independent *t* test. ^‡^BMI = body mass index = body weight (kg)/height^2^ (m^2^). ^§^NCT = noncontact intraocular pressure (noncontact tonometer).

## Data Availability

No data were used to support this study.
